# Adoption of preventive behaviors in response to the 2009 H1N1 influenza pandemic: a multiethnic perspective

**DOI:** 10.1111/irv.12306

**Published:** 2015-04-23

**Authors:** Gillian K SteelFisher, Robert J Blendon, Minah Kang, Johanna R M Ward, Emily B Kahn, Kathryn EW Maddox, Keri M Lubell, Myra Tucker, Eran N Ben-Porath

**Affiliations:** a Harvard T.H. Chan School of Public HealthBoston, MA, USA; b John F. Kennedy School of GovernmentCambridge, MA, USA; c Ewha Womans UniversitySeoul, Korea; d Centers for Disease Control and PreventionAtlanta, GA, USA; e Chenega Government Consulting, LLCChesapeake, VA, USA; f SSRS MediaPA, USA

**Keywords:** H1N1 subtype, health behavior, influenza A virus, pandemic, public opinion, race

## Abstract

**Background:**

As public health leaders prepare for possible future influenza pandemics, the rapid spread of 2009 H1N1 influenza highlights the need to focus on measures the public can adopt to help slow disease transmission. Such measures may relate to hygiene (e.g., hand washing), social distancing (e.g., avoiding places where many people gather), and pharmaceutical interventions (e.g., vaccination). Given the disproportionate impact of public health emergencies on minority communities in the United States, it is important to understand whether there are differences in acceptance across racial/ethnic groups that could lead to targeted and more effective policies and communications.

**Objectives:**

This study explores racial/ethnic differences in the adoption of preventive behaviors during the 2009 H1N1 influenza pandemic.

**Patients/Methods:**

Data are from a national telephone poll conducted March 17 to April 11, 2010, among a representative sample of 1123 white, 330 African American, 317 Hispanic, 268 Asian, and 262 American Indian/Alaska Native adults in the USA.

**Results:**

People in at least one racial/ethnic minority group were more likely than whites to adopt several behaviors related to hygiene, social distancing, and healthcare access, including increased hand washing and talking with a healthcare provider (*P*-values <0·05). Exceptions included avoiding others with influenza-like illnesses and receiving 2009 H1N1 and seasonal influenza vaccinations. After we controlled the data for socioeconomic status, demographic factors, healthcare access, and illness- and vaccine-related attitudes, nearly all racial/ethnic differences in behaviors persisted.

**Conclusions:**

Minority groups appear to be receptive to several preventive behaviors, but barriers to vaccination are more pervasive.

## Introduction

As a novel influenza A virus (H1N1) spread to more than 74 countries between March and mid-June, 2009, the World Health Organization declared a global pandemic.^1^ As public health leaders prepare for future influenza pandemics, the rapid spread of 2009 H1N1 influenza highlights the need to focus on measures that members of the public can adopt to help slow disease transmission. Influenza mitigation efforts have included practices related to hygiene (e.g., hand washing), social distancing (e.g., avoiding places where many people gather), and pharmaceutical interventions (e.g., vaccination).^2^–^7^ Increasing public acceptance of these preventive behaviors during a pandemic is a crucial goal for preparedness planning. Given the disproportionate impact of public health emergencies on minority communities in the United States,^8^–^10^ it is important to understand whether there are differences in acceptance across different racial/ethnic groups that could lead to targeted and more effective policies and communications across populations.

Evidence about racial/ethnic differences in behavioral responses to influenza, whether 2009 H1N1 or seasonal, is limited. Studies in the United States have focused primarily on vaccination uptake rather than use of antiviral medications, hand hygiene and respiratory etiquette practices, or social distancing behaviors.^11^ In most studies, vaccination rates for 2009 H1N1 or seasonal influenza appear to be higher among whites than African Americans or Hispanics, but few studies include discussion of American Indians/Alaska Natives or Asians.^11^–^14^ There is relatively little study of the reasons underlying differences in behavior related to 2009 H1N1 influenza. Available data suggest that reasons may be similar to those that pertain to differences in seasonal influenza vaccination rates,^15^ including those related to socioeconomic status, demographics, access to healthcare services, and attitudes (toward the vaccine, providers, and the illness).^12^–^17^

The limited data available on non-vaccine behaviors come from polling literature. Results from a poll regarding the American public's response to a hypothetical influenza pandemic suggest that African Americans may be less likely than whites to adopt financially burdensome social distancing behaviors, such as staying home from work for relatively long periods of time (e.g., 1 month).^18^ When considering less financially burdensome behaviors, however, African Americans are more likely to adopt them. Data from a poll on avian influenza suggest that racial/ethnic minorities are more concerned than whites about this illness and predict they would be more likely to take basic preventive actions, including washing hands more often, if avian influenza were detected in the USA population (R.J. Blendon, unpub. data). One might find similar racial/ethnic differences in non-vaccine behaviors for other infectious illnesses, like 2009 H1N1 influenza, but no related studies yet exist.

In this study, we used national polling data to explore racial/ethnic differences in the adoption of preventive behaviors related to hygiene, social distancing, and health care among adults in the United States during the 2009 H1N1 influenza pandemic. We also explored whether behavioral differences could be attributed to differences in socioeconomic status, demographic factors, access to healthcare services, or attitudes. Unlike many previous studies in the area of pandemic preparedness, this study assesses practices among American Indians/Alaska Natives and Asians, in addition to African Americans, Hispanics, and whites.

## Methods

### Design, sample, and data collection

Researchers at Harvard T.H. Chan School of Public Health (Boston, MA) conducted a telephone poll (landline and cell phone) from March 17 to April 11, 2010. A sample of the USA adult (≥18 years) population was identified using random-digit dialing to help ensure representativeness. Callbacks were staggered by time of day and day of week as well as systematic respondent selection within households. The sample included a total of 2355 adults. They self-identified as belonging to the following racial/ethnic groups: 1123 non-Hispanic whites (whites); 330 non-Hispanic African Americans (African Americans); 317 non-American Indian/Alaska Native Hispanics (Hispanics); 268 Asians; and 262 American Indians/Alaska Natives (AI/ANs). Interviews were conducted in English, Spanish, and Mandarin. Field operations were conducted by SSRS (Media, PA). The sample was weighted in two stages. First, each ethnic group was balanced to known demographic parameters (gender, age, race, education, region, and phone status) using the 2007 American Community Survey and 2008 National Health Interview Survey.^19^,^20^ Thus, known systematic differences between poll respondents and their ethnic group within the general population were addressed. The 2009 Current Population Survey was used to apply subsequent weights to the sample according to national racial/ethnic population distributions so that the total population makeup accurately reflected the proportion of each ethnic group among the general USA adult population.^21^

The study was completed as part of a cooperative agreement with the Centers for Disease Control and Prevention (CDC) and the National Public Health Information Coalition (NPHIC). It was part of a series designed to track behaviors and provide updated information to public health officials during the 2009 H1N1 influenza pandemic. For this reason, this study used polling methods for reaching target populations during a crisis. Polls have a relatively short field time (days or weeks) that can quickly provide public health officials with data for enhancing current policies.^22^,^23^ Although polls generally have lower response rates than longer term surveys (22% for this poll) and can have differential non-response rates across racial/ethnic groups, research suggests that data are comparable when polls are based on representative samples and re-weighted to key demographics, as described above.^24^,^25^ The study was deemed not human subjects research by the Harvard T.H. Chan School of Public Health Office of Human Research Administration.

### Polling instrument

The poll consisted of approximately 60 closed-ended questions about experiences with 2009 H1N1 influenza, attitudes about the illness and vaccine, and preventive behaviors [full question wording in appendix]. For analysis, behaviors were organized into three areas: (i) hygiene-related behaviors (e.g., washing hands, cleaning workspace or home); (ii) social distancing behaviors (e.g., avoiding places where many people gather, avoiding air travel); and (iii) healthcare-related behaviors (e.g., talking to a healthcare provider, receiving 2009 H1N1 influenza vaccine). We examined behaviors that were related to explicit recommendations by public health officials, as well as some that were not, to capture a relatively wide range of behaviors.^26^ We included a measure of seasonal influenza vaccination because public health officials promote its adoption during pandemics to reduce the total burden on the healthcare system and thus support population health; in this way, it may be considered a preventive measure even though it does not protect against the pandemic strain directly.^27^

### Analysis

In our primary analysis, we generated bivariate statistics to assess whether the outcomes of interest—adoption of self-protective behaviors (operationalized as “yes” to each behavior)—varied by racial/ethnic group. Race/ethnicity was defined by respondents’ identification with the following mutually exclusive groups: 1123 non-Hispanic whites (whites), 330 non-Hispanic African Americans (African Americans), 317 non-American Indian/Alaska Native Hispanics (Hispanics), 268 Asians, and 262 American Indians/Alaska Natives (AI/ANs). Whites served as the reference group. Two-tailed *t*-tests were used to assess statistical differences. Fifty-five people refused the questions that would have identified their race/ethnicity, and they were therefore not included in the racial/ethnic group analyses, although they are included in the total population estimates.

Secondarily, we examined whether other predictive variables might explain racial/ethnic differences in behaviors differed across racial/ethnic groups, again using two-tailed t-tests. We focused on variables suggested by the influenza vaccine literature^12^–^17^: (i) socioeconomic status, measured here by education (“high school degree or less,” some college or technical school, college degree, or more); (ii) demographic differences, including age (18–29, 30–49, 50–64, 65+ years, which reflect an approximation of time periods in the life cycle when attitudes and experiences with influenza-related prevented behaviors are likely different) and sex (male and female)^15^; (iii) healthcare system access, measured here by insurance status and employment (full time, part time, retired, or otherwise not employed)^28^; and (iv) attitudes including (1) concern “at any time since the beginning of the H1N1 outbreak in April 2009” that “you or a member of your family” would get 2009 H1N1 influenza and (2) views of 2009 H1N1 influenza vaccine safety. In addition, we measured other differences related to perceived risk of contracting a serious case of 2009 H1N1 influenza, including (a) being a parent of a child <18 years old who lives in the household (since children were targeted for vaccination based on higher complication risk) and (b) being at “high health risk,” defined as having at least one health issue (e.g., heart disease) that increases a person's risk for complications from influenza.^26^,^29^ All differences reported were significant at an alpha level of 0·05.

In the final step of analysis, we conducted logistic regression models for each behavior to determine whether observed bivariate differences in preventive behaviors across racial/ethnic groups persisted after we controlled the data for socioeconomic status, demographics, healthcare system access, and attitudes (as described above). We included only relevant predictive variables for each outcome. For example, we included attitudes toward the safety of the 2009 H1N1 influenza vaccine in the models about receiving the vaccine, but not in the models about hygiene-related behaviors. Given evidence from other studies that being in the “very concerned” as opposed to “somewhat concerned” category of attitudes was more predictive of preventive behaviors during the pandemic,^14^ we dichotomized both 4-point scale questions into variables that compared the “very” category (very concerned about 2009 H1N1 influenza infection or belief that the vaccine is very safe) to the remaining three categories combined (“somewhat,” “not very,” or “not at all” concerned/safe). Odds ratios (ORs) and 95% confidence intervals were estimated. We discuss the racial/ethnic variables and any others that were significant in models for all related behaviors (e.g., all hygiene-related behaviors). All analyses used SPSS v. 18 (IMB Corporation, Armonk, NY, USA) and accounted for weighted data.

## Results

### Racial/ethnic differences in preventive behaviors in response to the 2009 H1N1 influenza pandemic: uncontrolled comparisons

Racial/ethnic minority groups were more likely than whites to adopt many of the preventive behaviors, with differences between African Americans and whites being most common (Table1). However, the relative frequency (i.e., ranking) of behaviors was generally the same across racial/ethnic groups: Behaviors most commonly adopted were those related to hygiene, while social distancing behaviors (except avoiding someone with flu-like symptoms) and healthcare-related behaviors were less common.

**Table 1:** Percentage of persons reporting preventive behaviors against novel influenza A (H1N1) by racial/ethnic group: uncontrolled comparisons

Variables	All Respondents	White	African American	Hispanic	Asian	AI/AN∥
	% (*n* = 2355)	% (*n* = 1123)	% (*n* = 330)	% (*n* = 317)	% (*n* = 268)	%( *n* = 262)
Hygiene-related behaviors						
Washed hands more frequently*	82	80	**87**	86	86	81
Used hand sanitizer more frequently*	71	70	**81**	74	68	75
More frequently covered mouth and nose with tissue when coughing or sneezing*	67	63	**80**	**77**	70	79
More frequently coughed or sneezed into elbow or shoulder*	61	60	59	68	59	70
Tried to keep from touching eyes, nose, or mouth*	60	58	**68**	63	57	58
More frequently cleaned or disinfected home or workspace*	48	40	**66**	**69**	45	59
Used additional or stronger cleaners or disinfectants than normally used*	20	15	**33**	**37**	20	31
Social distancing behaviors						
Took any steps to avoid being near someone who has flu-like symptoms*	68	68	74	70	64	74
Avoided places where many people are gathered together*	19	15	21	**32**	**27**	**17**
Avoided air travel*^,^§	16	12	**20**	**30**	**21**	**28**
Limited use of public transportation, buses and trains*^,^¶	14	10	**22**	**29**	**18**	**23**
Healthcare-related behaviors						
Talked to doctor, nurse, or other health professional about what could be done to protect self or family from H1N1*	38	34	**47**	**48**	**43**	**47**
Recieved prescription for or purchased antivirals, such as Tamiflu or Relenza *	10	9	11	**18**	12	9
Took vitamins or herbal supplements beyond usual amount *	17	14	**25**	**29**	20	20
Recieved the H1N1 influenza vaccine for themselves since it became available in October 2009†	23	24	22	22	28	30
Recieved the seasonal influenza vaccine for themselves since September 2009‡	41	43	**30**	37	42	42

Findings in bold are statistically significantly different from whites at *P* < 0·05.

* % saying since the beginning of the H1N1 outbreak in April 2009, they have, at any point, done the following in response to H1N1.

† % saying they had recieved the H1N1 influenza vaccine since it first became available in October 2009.

‡ % saying they had recieved the seasonal influenza vaccine since September 2009.

§ Among % saying they traveled by air prior to H1N1.

¶ Among % saying used public transportation prior to H1N1.

∥ American Indian/Alaska Native.

#### Hygiene-related behaviors

Respondents in at least one racial/ethnic minority group were more likely than whites to adopt each of the hygiene-related behaviors. African Americans were more likely than whites to wash their hands more frequently (87% African Americans versus 80% whites), sanitize their hands more frequently (81% versus 70%), and “try to keep from touching eyes, nose, or mouth” (68% versus 58%). All racial/ethnic minorities except Asians were more likely than whites to cover coughs and sneezes “with a tissue” more frequently (80% African Americans, 77% Hispanics, and 79% AI/ANs versus 63% whites), and American Indian/Alaska Natives were also more likely than whites to cough/sneeze “into [their] elbow or shoulder” more frequently (70% versus 60%). All racial/ethnic minorities except Asians were more likely than whites to clean/disinfect their “home or workspace” more frequently (66% African Americans, 69% Hispanics, and 59% AI/ANs versus 40% whites) and to use stronger cleaners (33% African Americans, 37% Hispanics, and 31% AI/ANs versus 15% whites).

#### Social distancing measures

Between 64% and 74% of each racial/ethnic group said they had “taken any steps to avoid being near someone who has flu-like symptoms,” with no statistical differences between any minority group and whites. Among people who normally travel by air or use public transportation, people in all racial/ethnic minority groups were more likely than whites to say they “avoided air travel” or “limited [their] use of public transportation, buses and trains,” while Hispanics and Asians were also more likely than whites to say they “avoided places where many people gather” (32% Hispanics and 27% Asians versus 15% whites).

#### Healthcare-related behaviors

Nearly half of respondents in all racial/ethnic minority groups said they “talked to a doctor, nurse, or other health professional about protecting themselves or family from H1N1” compared with one-third for whites (47% African Americans, 48% Hispanics, 47% AI/ANs, and 43% Asians versus 34% whites). Other healthcare-related behaviors were less common, and there were fewer differences in behaviors between groups. Hispanics were more likely than whites to get a prescription for antiviral medications (18% versus 9%), while both African Americans and Hispanics were more likely to say they had “taken vitamins or herbal supplements beyond the usual amount [they normally] take” (25% African Americans and 29% Hispanics versus 14% whites). Getting the 2009 H1N1 influenza vaccine did not differ significantly between racial/ethnic groups, but African Americans were less likely than whites to get the seasonal influenza vaccine (30% versus 43%).

Racial/ethnic differences in predictive variables that might explain behavioral differences: socioeconomic status, demographics, access to health care, and attitude-related variables: uncontrolled comparisons 2.

There was variation across racial/ethnic groups with respect to socioeconomic status, age, and access to healthcare services. All racial/ethnic groups were more likely to have lower levels of education than whites, with the exception of Asians (Table2). For example, African Americans, Hispanics, and American Indians/Alaska Natives were more likely than whites to have a “high school degree or less” (53% African Americans, 68% Hispanics, and 59% AI/ANs versus 41% whites). White respondents were more likely than all racial/ethnic minority groups to be ≥ 65 years old (13% African Americans, 9% Hispanics, 12% AI/ANs, and 12% Asians versus 19% whites). Furthermore, except for Asians, respondents in racial/ethnic minorities were less likely to have health insurance than whites (76% African Americans, 58% Hispanics, and 77% AI/ANs versus 85% whites), and American Indians/Alaska Natives were less likely than whites to be employed full time (34% versus 44%).

**Table 2:** Racial/ethnic differences in factors that might explain behavioral differences: socioeconomic status, demographics, access to health care, and attitude-related variables

Variables	All Respondents	White	African American	Hispanic	Asian	AI/AN*
	% (*n* = 2355)	% (*n* = 1123)	% (*n* = 330)	% (*n* = 317)	% (*n* = 268)	% (*n* = 262)
Socioeconomic status						
Education						
High school degree or less	45	41	**53**	**68**	32	59
Some college/tech school	30	33	32	**22**	**24**	30
College degree or more	27	29	**17**	**12**	46	**12**
Demographics						
Age						
18–29 years	20	18	**25**	**30**	21	23
30–49 years	38	36	38	**45**	**45**	39
50–64 years	25	27	23	17	22	26
65 years or older	17	19	**13**	**9**	**12**	**12**
Sex						
Male	48	49	43	50	47	48
Female	52	51	57	50	53	52
Access to health care						
Health insurance (covered)	81	85	**76**	**58**	87	**77**
Employment status						
Employed full time	43	44	42	41	45	**34**
Employed part time	12	12	11	12	13	14
Not employed	44	44	47	46	42	52
Attitude-related variables						
Concern about whether they or someone in their immediate family would get sick from H1N1 since the beginning of the H1N1 outbreak in April 2009 (% saying they were…)						
Very concerned	20	16	**33**	**29**	**21**	**24**
Somewhat concerned	27	31	**14**	**20**	**26**	**16**
Not very concerned	4	4	3	5	5	4
Not at all concerned	49	49	49	47	47	55
Belief that H1N1 vaccine is, in general for most people,…						
Very safe	34	37	**21**	29	33	**28**
Somewhat safe	43	43	47	42	42	**59**
Not very safe	9	8	10	**17**	9	**1**
Not at all safe	4	3	**8**	6	6	7
Parental status (yes)	33	31	32	**44**	**42**	34
Health problems associated with influenza complications (yes)	21	21	21	19	**11**	**31**

Findings in bold are statistically significantly different from whites at *P* < 0·05.

* American Indian/Alaska Native.

Attitudes about the 2009 H1N1 influenza virus and vaccine safety varied between racial/ethnic minority groups and whites. African Americans, Hispanics, and American Indians/Alaska Natives were all more likely than whites to say they were “very concerned” about themselves or a member of their family getting sick with 2009 H1N1 influenza during the pandemic (33% African Americans, 29% Hispanics, and 24% AI/ANs versus 16% whites). Respondents in some racial/ethnic minorities were also more likely than whites to have characteristics associated with perceived risk. Compared with whites, Hispanics and Asians were more likely to be parents (44% Hispanics and 42% Asians versus 31% whites). American Indians/Alaska Natives were more likely than whites to report health problems associated with influenza complications, although Asians were less likely to do so (31% AI/ANs versus 21% Asians versus 11% whites). African Americans and American Indians/Alaska Natives were less likely than whites to say they believed the 2009 H1N1 influenza vaccine was “very safe” (28% African Americans and 21% AI/ANs versus 37% whites).

### Racial/ethnic differences in behaviors after the logistic regression controlled for predictive variables that might explain behavioral differences

#### Hygiene-related behaviors

After we controlled the data for socioeconomic, demographic, relevant healthcare, and relevant attitude-related variables, many of the bivariate differences persisted, except for differences in more frequent hand washing (Table3A–C). African Americans had higher odds than whites of using hand sanitizer more frequently (OR 1·73). All racial/ethnic minority groups had higher odds than whites of covering sneezes and coughs with a tissue more frequently (African Americans OR 2·24; Hispanics OR 1·87; AI/ANs OR 2·14; Asians OR 1·44), while only American Indians/Alaska Natives had higher odds than whites of sneezing/coughing into their elbow or shoulder more frequently (OR 1·54). African Americans had higher odds than whites for trying to keep from touching their eyes, nose, or mouth (OR 1·45). All racial/ethnic minority groups, except Asians, had higher odds than whites of cleaning/disinfecting their homes or workspaces more frequently (African Americans OR 2·32; Hispanics OR 2·57; AI/ANs OR 1·74), and all racial/ethnic groups had higher odds than whites of using additional or stronger cleaners (African Americans OR 2·22; Hispanics OR 2·39; AI/ANs OR 1·99; Asians OR 1·53). Regardless of race/ethnicity, respondents who were very concerned about contracting 2009 H1N1 influenza had higher odds than others of adopting all the hygiene behaviors, and women had higher odds than men of adopting all behaviors except using additional/stronger cleaners.

**Table 3:** (A) Racial/ethnic differences in hygiene-related behaviors after controlling for factors that might explain the differences

Variables	Washed hands more frequently		Used hand sanitizer more frequently		More frequently covered mouth and nose with tissue when coughing or sneezing		More frequently coughed or sneezed into elbow or shoulder		Tried to keep from touching eyes, nose, or mouth		More frequently cleaned or disinfected home or workspace		Used additional or stronger cleaners or disinfectants than normally used	
	OR	95% CI	OR	95% CI	OR	95% CI	OR	95% CI	OR	95% CI	OR	95% CI	OR	95% CI
Race/ethnicity														
White (reference)														
African American	1·30	(0·89,1·90)	**1·73**	**(1·25,2·39)**	**2·24**	**(1·63,3·09)**	0·86	(0·66,1·13)	**1·45**	**(1·10,1·90)**	**2·32**	**(1·76,3·05)**	**2·22**	**(1·65,2·99)**
Hispanic	1·19	(0·83,1·72)	1·10	(0·81,1·48)	**1·87**	**(1·37,2·55)**	1·32	(0·99,1·76)	1·14	(0·87,1·50)	**2·57**	**(1·94,3·41)**	**2·39**	**(1·78,3·22)**
Asian	1·45	(0·98,2·15)	0·93	(0·69,1·26)	**1·44**	**(1·06,1·95)**	0·89	(0·66,1·19)	0·94	(0·71,1·24)	1·12	(0·84,1·50)	**1·53**	**(1·07,2·19)**
American Indian/Alaska Native	0·86	(0·60,1·23)	1·17	(0·85,1·62)	**2·14**	**(1·52,3·02)**	**1·54**	**(1·13,2·09)**	0·93	(0·70,1·23)	**1·74**	**(1·30,2·33)**	**1·99**	**(1·43,2·75)**
Education														
High school graduate or less (reference)														
Some college/tech school	0·99	(0·76,1·30)	0·99	(0·78,1·24)	**0·60**	**(0·48,0·75)**	1·18	(0·95,1·46)	0·86	(0·70,1·06)	**0·75**	**(0·61,0·93)**	**0·51**	**(0·40,0·65)**
College degree or more	0·91	(0·68,1·21)	1·01	(0·79,1·30)	**0·76**	**(0·59,0·97)**	1·19	(0·94,1·49)	0·98	(0·78,1·22)	**0·64**	**(0·51,0·81)**	**0·43**	**(0·32,0·57)**
Age														
18–29 years (reference)														
30–49 years	1·12	(0·82,1·54)	1·02	(0·78,1·33)	**1·42**	**(1·10,1·84)**	1·00	(0·78,1·28)	1·03	(0·81,1·31)	0·88	(0·68,1·12)	**0·71**	**(0·54,0·93)**
50–64 years	1·07	(0·77,1·50)	1·17	(0·88,1·57)	**1·68**	**(1·26,2·23)**	0·93	(0·71,1·22)	1·10	(0·85,1·43)	0·92	(0·71,1·21)	**0·67**	**(0·49,0·92)**
65 years or older	0·75	(0·52,1·08)	**0·68**	**(0·49,0·94)**	**1·73**	**(1·24,2·40)**	0·81	(0·60,1·10)	0·92	(0·68,1·24)	**0·47**	**(0·34,0·64)**	**0·51**	**(0·35,0·75)**
Sex														
Male (reference)														
Female	**1·82**	**(1·44,2·29)**	**1·28**	**(1·06,1·55)**	**1·49**	**(1·23,1·81)**	**1·80**	**(1·51,2·16)**	**1·44**	**(1·21,1·71)**	**1·69**	**(1·41,2·02)**	1·22	(0·99,1·51)
Concern about self/family getting sick with H1N1														
Somewhat/not very/not concerned (reference)														
Very concerned	**3·91**	**(2·58,5·92)**	**2·39**	**(1·81,3·16)**	**2·76**	**(2·07,3·67)**	**1·78**	**(1·40,2·25)**	**2·10**	**(1·67,2·63)**	**2·64**	**(2·10,3·33)**	**2·19**	**(1·74,2·77)**
Parental status														
Not parent (reference)														
Parent	1·20	(0·91,1·59)	0·86	(0·68,1·08)	0·92	(0·73,1·15)	**1·33**	**(1·07,1·64)**	0·84	(0·68,1·03)	1·07	(0·87,1·32)	1·04	(0·82,1·33)
Health issues associated with influenza complications														
No health issue (reference)														
Health issue	1·28	(0·95,1·73)	1·28	(1·00,1·63)	**1·37**	**(1·07,1·76)**	0·95	(0·76,1·19)	1·04	(0·84,1·29)	**1·25**	**(1·00,1·57)**	**1·33**	**(1·04,1·71)**

Variables	Took steps to avoid being near someone who has flu-like symptoms		Avoided places where many people are gathered together		Avoided air travel*		Limited use of public transportation, buses and trains*	
	OR	95% CI	OR	95% CI	OR	95% CI	OR	95% CI
(B) Racial/ethnic differences in social distancing								
Race/ethnicity								
White (reference)								
African American	1·13	(0·84,1·51)	1·26	(0·90,1·76)	**1·53**	**(1·08,2·18)**	**2·00**	**(1·40,2·86)**
Hispanic	0·95	(0·71,1·26)	**2·37**	**(1·73,3·23)**	**2·32**	**(1·67,3·23)**	**2·61**	**(1·86,3·68)**
Asian	0·80	(0·59,1·07)	**2·34**	**(1·67,3·28)**	**2·39**	**(1·63,3·49)**	**2·14**	**(1·43,3·20)**
American Indian/Alaska Native	1·23	(0·89,1·69)	0·97	(0·66,1·42)	**2·21**	**(1·55,3·16)**	**1·88**	**(1·28,2·76)**
Education								
High school degree or less (reference)								
Some college/tech school	**1·29**	**(1·03,1·61)**	**0·56**	**(0·43,0·74)**	**0·50**	**(0·38,0·66)**	**0·46**	**(0·34,0·62)**
College degree or more	1·02	(0·80,1·29)	**0·67**	**(0·50,0·89)**	**0·29**	**(0·21,0·41)**	**0·47**	**(0·33,0·66)**
Age								
18–29 years (reference)								
30–49 years	1·03	(0·79,1·33)	1·04	(0·76,1·42)	0·98	(0·72,1·33)	**0·62**	**(0·45,0·85)**
50–64 years	0·93	(0·71,1·23)	1·24	(0·89,1·74)	0·81	(0·57,1·15)	0·75	(0·52,1·07)
65 years or older	0·80	(0·58,1·09)	1·41	(0·96,2·07)	0·69	(0·45,1·05)	0·78	(0·51,1·18)
Sex								
Male (reference)								
Female	**1·45**	**(1·20,1·74)**	0·87	(0·70,1·09)	**0·74**	**(0·58,0·93)**	**0·70**	**(0·55,0·89)**
Concern about self/family getting sick with H1N1								
Somewhat/not very/not concerned (reference)								
Very concerned	**3·30**	**(2·49,4·38)**	**2·99**	**(2·35,3·79)**	**2·93**	**(2·27,3·77)**	**2·97**	**(2·28,3·86)**
Parental status								
Not parent (reference)								
Parent	1·09	(0·87,1·37)	1·06	(0·81,1·37)	0·98	(0·75,1·29)	1·23	(0·92,1·63)
Health issues associated with influenza complications								
No health issue (reference)								
Health issue	**1·31**	**(1·03,1·66)**	**1·67**	**(1·29,2·15)**	**1·71**	**(1·30,2·24)**	**1·45**	**(1·09,1·92)**

Variables	Talked to doctor, nurse, or other health professional about what could be done to protect self or family from H1N1		Received prescription for or purchased antivirals, such as Tamiflu or Relenza		Took vitamins or herbal supplements beyond usual amount		Received H1N1 vaccine		Received seasonal influenza vaccine	
	OR	95% CI	OR	95% CI	OR	95% CI	OR	95% CI	OR	95% CI
(C) Racial/ethnic differences in healthcare										
Race/ethnicity										
White (reference)										
African American	**1·38**	**(1·05,1·82)**	0·95	(0·63,1·46)	**1·65**	**(1·20,2·27)**	1·06	(0·75,1·50)	**0·58**	**(0·43,0·78)**
Hispanic	**1·41**	**(1·06,1·87)**	**1·87**	**(1·27,2·75)**	**2·18**	**(1·59,3·00)**	1·03	(0·72,1·47)	1·16	(0·87,1·56)
Asian	1·29	(0·96,1·72)	1·17	(0·75,1·82)	**1·44**	**(1·01,2·06)**	**1·44**	**(1·03,2·03)**	1·07	(0·80,1·44)
American Indian/Alaska Native	**1·47**	**(1·09,1·97)**	0·84	(0·51,1·36)	1·43	(1·00,2·04)	**1·57**	**(1·11,2·22)**	1·07	(0·79,1·45)
Education										
High school graduate or less (reference)										
Some college/tech school	1·03	(0·83,1·28)	0·96	(0·69,1·34)	1·03	(0·80,1·34)	1·14	(0·88,1·49)	0·92	(0·73,1·15)
College degree or more	1·11	(0·87,1·42)	0·85	(0·59,1·23)	0·97	(0·72,1·31)	1·10	(0·83,1·46)	1·25	(0·98,1·59)
Age										
18–29 years (reference)										
30–49 years	0·92	(0·72,1·18)	0·90	(0·63,1·30)	0·96	(0·71,1·29)	0·85	(0·62,1·16)	1·23	(0·94,1·61)
50–64 years	0·78	(0·59,1·04)	**0·60**	**(0·38,0·94)**	1·00	(0·71,1·39)	0·79	(0·56,1·12)	**1·98**	**(1·48,2·65)**
65 years or older	0·78	(0·51,1·18)	**0·40**	**(0·20,0·80)**	0·78	(0·47,1·30)	1·01	(0·62,1·62)	**3·76**	**(2·48,5·70)**
Sex										
Male (reference)										
Female	1·03	(0·86,1·24)	1·08	(0·81,1·43)	1·15	(0·92,1·44)	1·10	(0·88,1·37)	1·16	(0·96,1·40)
Insurance status										
Not covered by health insurance (reference)										
Covered by health insurance	1·08	(0·85,1·38)	**1·63**	**(1·11,2·40)**	0·81	(0·61,1·07)	**1·51**	**(1·1,2·09)**	**2·48**	**(1·88,3·27)**
Employment										
Employed full time (reference)										
Employed part time	1·17	(0·87,1·57)	1·02	(0·65,1·59)	1·10	(0·77,1·56)	0·92	(0·63,1·33)	0·94	(0·69,1·28)
Retired	1·11	(0·77,1·60)	1·66	(0·93,2·94)	1·35	(0·87,2·07)	**1·52**	**(1·01,2·29)**	1·35	(0·94,1·92)
Not employed	**1·33**	**(1·06,1·68)**	0·92	(0·65,1·30)	1·04	(0·79,1·37)	1·30	(0·98,1·71)	1·05	(0·83,1·33)
Concern about self/family getting sick with H1N1										
Somewhat/not very/not concerned (reference)										
Very concerned	**3·38**	**(2·71,4·22)**	**2·68**	**(2·00,3·60)**	**1·91**	**(1·49,2·43)**	**2·13**	**(1·66,2·74)**	**1·84**	**(1·47,2·30)**
Belief in H1N1 vaccine safety										
Somewhat/not very/not at all safe (reference)										
Very safe	—	—	—	—	—	—	**5·81**	**(4·66,7·24)**	—	—
Parental status										
Not parent (reference)										
Parent	**1·83**	**(1·48,2·26)**	**1·42**	**(1·04,1·94)**	0·92	(0·71,1·19)	1·25	(0·96,1·62)	1·11	(0·89,1·39)
Health issues associated with influenza complications										
No health issue (reference)										
Health issue	**1·68**	**(1·35,2·10)**	1·14	(0·82,1·59)	1·01	(0·77,1·31)	**1·60**	**(1·24,2·05)**	**1·86**	**(1·49,2·33)**

Findings in bold are statistically significantly different from whites at *P* < 0·05.

* Regressions conducted among those who routinely travel by air or by public transportation, respectively.

#### Social distancing

Among those who normally travel by air or use public transportation, all minority groups had higher odds than whites of avoiding air travel (African Americans OR 1·53; Hispanics OR 2·32; AI/ANs OR 2·21; and Asians OR 2·39) or limit their use of public transportation (African Americans OR 2·00; Hispanics OR 2·61; AI/ANs OR 1·88; Asians OR 2·14), after we controlled the data for socioeconomic, demographic, healthcare, and attitude-related variables (Table3B). Hispanics and Asians also had higher odds than whites of avoiding places where many people gather (Hispanics OR 2·37, Asians OR 2·34). Regardless of race/ethnicity, those with a high risk for influenza complications and those who were very concerned about contracting 2009 H1N1 influenza had higher odds than others of adopting all social distancing behaviors. By contrast, those with more education had higher odds than others adopting all social distancing behaviors.

#### Healthcare-related behaviors

After we controlled the data for demographic, healthcare access, and attitude-related variables, racial/ethnic differences in behaviors persisted (Table3C). All racial/ethnic minorities, except Asians, had higher odds than whites of talking with a healthcare provider (African Americans OR 1·38; Hispanics OR 1·41; AI/ANs OR 1·47). All racial/ethnic groups, except American Indians/Alaska Natives, had higher odds than whites of taking extra vitamins or herbal supplements (African Americans OR 1·65; Hispanics OR 2·18; Asians OR 1·44). Hispanics had higher odds than whites of obtaining a prescription for antiviral medications (OR 1·87). American Indians/Alaska Natives (OR 1·57) and Asians (OR 1·44) had higher odds than whites of receiving the 2009 H1N1 influenza vaccine. African Americans had higher odds than whites to get the seasonal influenza vaccine (OR 0·58). Regardless of race/ethnicity, respondents who were very concerned about contracting 2009 H1N1 influenza had higher odds than others of adopting all healthcare-related behaviors. Those who believed the 2009 H1N1 influenza vaccine was very safe had higher odds than others of receiving it.

## Discussion

This study provides important insights regarding racial/ethnic differences in the adoption of preventive behaviors related to hygiene, social distancing, and health care during the 2009 H1N1 influenza pandemic. Not all of these measures were recommended by public health authorities, but they reflect a selection of behaviors that members of the public adopted. Our central (uncontrolled) findings suggest that African Americans, Hispanics, and American Indians/Alaska Natives were more likely than whites to adopt most of these preventive behaviors. Compared with whites, Asians were more likely to adopt several social distancing measures and to talk to a health professional about 2009 H1N1 influenza, but their adoption of behaviors was otherwise similar to that of whites. Notably, none of the behaviors asked about in this poll are likely to be considered to be very burdensome, especially compared with behaviors related to workplace closure,^18^ and none were mandated by government. Thus, our study suggests receptivity in these racial/ethnic minority communities to adopting individual-level behaviors of this kind.

In contrast to other behaviors in this study, the primary analyses in this study show that racial/ethnic minorities were not more likely than whites to get the 2009 H1N1 influenza vaccine. Furthermore, African Americans were less likely than whites to get the seasonal flu vaccine. Our data do not identify disparities in 2009 H1N1 influenza vaccination rates between African Americans and whites as does another study, and it may be considered a success that racial/ethnic groups, such as American Indian/Alaska Native populations, received vaccines at a statistically equivalent rate to whites.^30^–^32^ However, the contrast between getting vaccinated and adopting other behaviors is nonetheless striking. The factors that motivate people in racial/ethnic minorities to adopt other preventive behaviors at rates greater than whites are not sufficient to overcome barriers to vaccination and may differ from those that motivate vaccination. Further, the overall vaccination rates are not high in any group,^11^,^32^ suggesting that barriers are prevalent across all racial/ethnic groups, even if the barriers are different.^33^ Public health officials should try to address underlying differences in motivation and barriers across racial/ethnic populations.

Factors that are considered primary reasons for vaccine-related disparities – socioeconomic status, demographic characteristics, access to health care, and attitudes^5^–^17^ – appear to play a role in racial/ethnic differences in the adoption of additional behaviors examined here, insofar as some racial/ethnic differences were eliminated once these variables were controlled for. However, many differences between racial/ethnic groups in the adoption of preventive behaviors persisted even after these controls, suggesting that other factors are likely playing a role in the differences between racial/ethnic groups. Literature from disaster preparedness suggests that differential trust in government and communication sources play a role in people's response to public health recommendations, particularly for racial/ethnic minorities.^8^,^10^,^34^–^37^ These factors may be important in the area of infectious disease emergencies as well and may partially explain racial/ethnic differences in response to H1N1. Other cultural and social factors that vary across racial/ethnic groups, including religious beliefs or health-related values, may also shape differences between racial/ethnic groups in the adoption of these measures. Future research is needed to explore these factors.

Several factors aside from race/ethnicity also appear to contribute to the adoption of many of the behaviors. These factors include health status (i.e., a higher risk of influenza complications) and gender, which were significant in the final models for a majority of behaviors. Moreover, attitudes toward the illness play a role in adoption of all behaviors, and perceptions of vaccine safety play a role in vaccine adoption. Finally, data suggest attitudes about the illness and the vaccine vary across racial/ethnic groups, indicating a need for public health officials to address attitudes in pandemic planning. Additional research to better understand the ways in which attitudes, including risk perceptions of the illness and vaccines, impact the adoption of preventive behaviors is warranted.

This study has limitations. First, the study was conducted in English, Spanish, and Mandarin, but not in other languages; thus, views of groups who speak other languages were not represented. Second, there was the potential for differential non-response bias across racial/ethnic subgroups that may not be fully addressed through weighting techniques; however, the magnitude of differences in non-response across racial/ethnic groups is likely small, and weighting corrections within the groups should render it unlikely; this accounts for the differences in reported behaviors between groups. Third, making multiple comparisons in a given analysis could, in theory, result in finding more statistically significant racial/ethnic differences than truly exist; however, the differences between racial/ethnic groups we did find are generally so large and repeated, so clearly across many groups, problems from multiple comparisons are unlikely to have played a meaningful role in the conclusions we draw from the data. Fourth, there were a small number of people who refused the questions about race/ethnicity, but they are unlikely to all have been of one race/ethnicity and to have different behavioral practices, and their absence from analysis is thus unlikely to have biased the results. Last, the variables we used as control measures may not fully account for the underlying construct. For example, health insurance and employment status may not fully measure access to vaccination, as they may not capture access to public health clinics. In such cases, these factors could play an even greater role in behavior adoption than we were able to evaluate in our study. As none of these limitations fundamentally alter the key findings of this study, results nonetheless provide direction for public health officials and others interested in increasing adoption of preventive measures during a pandemic.

## Authoship detalils

Gillian SteelFisher and Robert Blendon conceived of the study design, developed the study instrument, oversaw data collection, conducted analyses and interpretation of the data, and drafted the manuscript. Minah Kang developed the analysis plan, conducted statistical analyses, and participated in the writing and revision of the manuscript. Johanna Ward participated in developing the study instrument, data analysis/interpretation, and the writing of the manuscript. Emily Kahn, Kathryn Maddox, Myra Tucker, and Keri Lubell contributed to the interpretation of the data and provided subject matter expertise; they also contributed to the writing and revision of the manuscript. Eran Ben-Porath oversaw the operations of data collection, conducted some statistical analyses, and contributed to the writing and revisions of the manuscript. All authors read and approved the final version of the manuscript.
